# Supplementary material for: The Association between Smoking Cessation and Depressive Symptoms: Diet Quality Plays a Mediating Role
Source: Nutrients. 2022 Jul 25;14(15):3047. doi: 10.3390/nu14153047 (PMC9331914; doi:10.3390/nu14153047)
Supplement: Supplementary file 1 [file nutrients-14-03047-s001.zip › nutrients-1746780-supplementary.pdf]

Table S1. The classifications of covariates

Covariates	Classifications
**Gender**	Female; Male
**Age (year)**	Continuous
**Race**	Mexican American; Non-Hispanic White; Non-Hispanic Black; Other Hispanic; Other race
**Annual household income**	< 20000$, ⩾20000$
**Educational level**	Below high school; High school; Above high school;
**Body mass index**	Continuous
**Smoked at least 100 cigarettes in life**	No; Yes
**Had at least 12 alcohol drinks/year**	No; Yes
**Hypertension**	No; Yes
**Diabetes**	No; Yes
**Work physical activity**	Moderate; Vigorous; Other
**Recreational physical activity**	Moderate; Vigorous; Other
**Total energy intake (kcal/d)** [a]	Continuous
**Caffeine intake (mg/d)** [a]	Continuous

[a] the mean dietary intake through two 24-h dietary data.

Table S2. Association Between Smoking Cessation and Depressive Symptoms in model2, stratified by

HEI: NHANES 2007–2014 (N=20,004)

**HEI groups**	**<50**		**50-70**		**>=70**		***P*-interaction**
	OR	95% CI	OR	95% CI	OR	95% CI	
**Time Since Quit (years)**	0.974*	0.952-0.996	0.980*	0.960-0.999	1.031	0.997-1.037	0.0471
**Smoking status**							
**Current smokers**	1	(ref)	1	(ref)	1	(ref)	
**Time Since Quit (years)**							
**< 10 years**	0.642*	0.455-0.905	0.603*	0.401-0.909	0.778	0.243-2.481	
**10 – 20 years**	0.376**	0.236-0.599	0.304**	0.184-0.504	0.768	0.199-2.967	
**20 – 30 years**	0.611	0.306-1.220	0.496*	0.274-0.896	0.789	0.234-2.645	
**>=30 years**	0.384*	0.178-0.832	0.282**	0.136-0.584	1.547	0.435-5.510	
**Never smoked**	0.432**	0.341-0.547	0.429**	0.317-0.580	0.417*	0.195-0.894	

Calculated using binary logistic regression. Model 2 adjusted for age, gender, race/ethnicity, educational level, household income, caffeine intake, total energy intake, alcohol consumption, work activity, recreational activity, diabetes, hypertension and BMI.

*P < 0.05; **P < 0.01

Table S3. Baseline characteristics of participants by completeness of the depression scale, NHANES 2007–2014 (N=23352)

	Complete depression data	Missing depression data	P value
Number of participants (%) [a]	20,004 (85.66)	3348 (14.34)	
Age	49 (34,64)	47 (34,65)	0.85
Age (year) [a]			<0.001
18-39	6714 (35.91)	1206 (41.43)	
40-59	6590 (38.36)	1082 (36.63)	
≥60	6700 (25.72)	1060 (21.94)	
Gender (%) [a]			<0.001
Male	10007 (49.59)	1485 (43.32)	
Female	9997 (50.41)	1863 (56.68)	
Race/ethnicity (%) [a]			<0.001
Mexican American	2967 (8.27)	511 (9.82)	
Other Hispanic	2027 (5.37)	344 (6.46)	
Non-Hispanic White	8949 (68.76)	1206 (58.15)	
Non-Hispanic Black	4206 (11.03)	761 (13.92)	
Other races	1855 (6.58)	526 (11.65)	
Educational level (%) [a]			< 0.001
< high school	1990 (5.24)	564 (10.71)	
High school	7522 (34.24)	1288 (36.80)	
> high school	10159 (60.52)	1455 (52.50)	
Smoke status (%) [a]			<0.001
Never smoker	10962 (55.07)	1960 (57.68)	
Former smoker	4786 (24.27)	689 (19.78)	
Current smoker	4255 (20.67)	697 (22.54)	
Healthy Eating Index [b]	53.12 (43.97,62.76)	52.95 (44.45,62.87)	0.5678
Healthy Eating Index (%) [a]			0.7991
<50	7971 (41.77)	645 (41.45)	
50-70	9000 (45.88)	722 (45.44)	
≥70	2372 (12.35)	194 (13.11)	

Data are number of participants (weighted percentage) or medians (interquartile ranges).

PHQ, Patient Health Questionnaire; RDA, Recommended Dietary Allowance.

[a] Chi-square test was used to compare the percentage between participants with and without depression symptoms.

[b] Mann-Whitney U test was used to compare the difference between participants with and without depression symptoms.

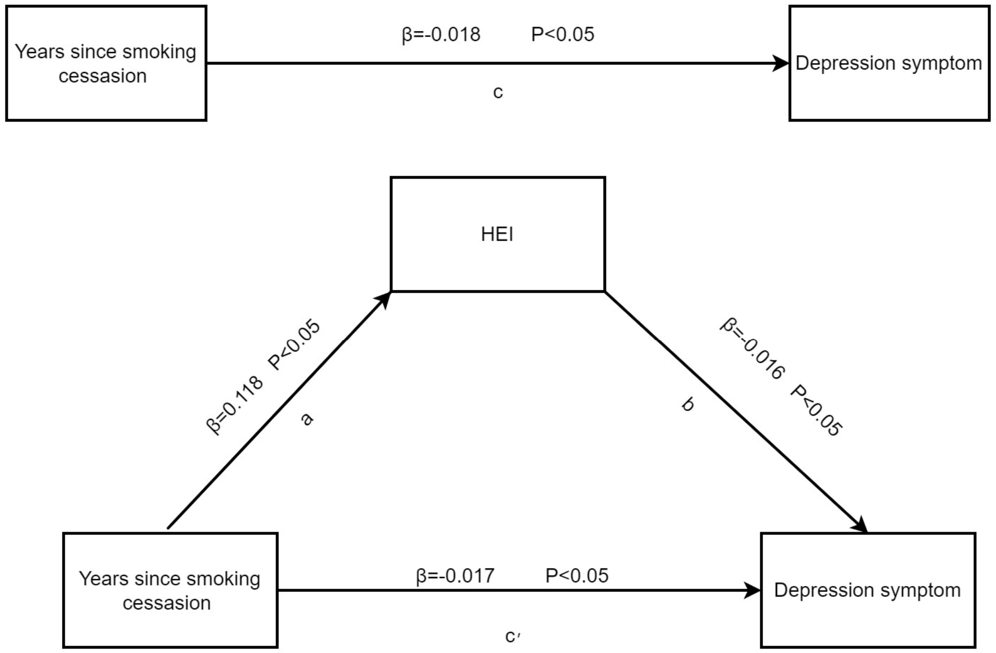

Figure S1: Diet quality mediate the relationship between Smoking cessation and depressive symptoms
